# Sialochemical analysis in polytraumatized patients in intensive care units

**DOI:** 10.1371/journal.pone.0222974

**Published:** 2019-10-03

**Authors:** Maria Heloisa Madruga Chaves, Amanda Rebeca da Silveira Wolf, Kelly Aline Lima Nascimento, Danielle Nawcki, Gabriele Muller Feustel, Patricia Vida Cassi Bettega, Sergio Aparecido Ignacio, João Armando Brancher, Luana Alves Tannous, Renata Iani Werneck, Paulo Henrique Couto Souza, Marlene Maria Tourais de Barros, Aline Cristina Batista Rodrigues Johann

**Affiliations:** 1 School of Life Sciences, Department of Nursing, Pontifícia Universidade Católica do Paraná, Curitiba, Paraná, Brazil; 2 School of Life Sciences, Department of Dentistry, Pontifícia Universidade Católica do Paraná, Curitiba, Paraná, Brazil; 3 School of Medicine, Pontifícia Universidade Católica do Paraná, Curitiba, Paraná, Brazil; 4 Instituto Ciências da Saúde, Universidade Católica Portuguesa-Viseu, Viseu, Portugal; Emergency University County Hospital ”Pius Brinzeu” Timisoara, ROMANIA

## Abstract

The profiles of polytraumatized patients in intensive care units were characterized. Serum and salivary markers were compared with normality between Classes I and II of APACHE II and between periods of hospitalization; these results were correlated. This was a prospective study on saliva charts and collection (n = 70). Profile: male, 27 years old, blunt traumas and collisions. Serum parameters with normality: decrease in pH, creatinine at admission to Class I, and at 48 and 72 hours in both classes; K^+^ at 48 h in Class II; Ca+ on admission in both classes and at 72 h in Class I. Increase in urea at 72 h in Class II, glucose at all times and in all classes, and Ca+ at 48 h in both classes. Class II had high Na^+^ at 48 and 72 h compared to Class I. In Class I, creatinine reduction occurred in 48 h and 72 h compared to admission and an increase of Ca+ at 48 h with admission. In Class II, pH and Na^+^ increased at 48 h and 72 h compared to admission. K^+^ decreased from admission to 48 h and increased from 48 h to 72 h. Urea increased from 48 to 72 hours. Creatinine decreased from admission to 48 and 72 hours. Ca+ increased from admission to 48 hours and decreased from 48 to 72 hours. There was an increase in the saliva levels in both classes and times in relation to normality. There was an increase in urea at admission, glucose at 72 h, and Ca+ at 48 h in Class II compared with Class I. Class I urea increased from admission to 48 h and Ca+ decreased from admission to 48 h. Class II urea decreased from 48 h to 72 h. Strong or very strong positive correlation was identified between blood and creatinine saliva at all times and regular and negative Ca+ at 72 h. This study provides evidence that salivary and serum biomarkers can be used together to monitor the evolution of the clinical symptoms of ICU patients.

## Introduction

According to the World Health Organization (WHO), 5.8 million people die each year from trauma worldwide, surpassing by 32% the deaths caused by malaria, AIDS and tuberculosis combined, which corresponds to 10% of all causes of death. Without due intervention, it is anticipated that this incidence will increase until 2030, dramatically raising personal and social costs. Traumas also account for a majority of permanent disabilities and are responsible for considerable economic losses to victims, their families, and the countries in general [1–3].

A trauma is defined as "one or more lesions, of varying extent, intensity and severity, which may be produced by various agents (physical, chemical, electrical), either accidentally or intentionally, capable of producing local or systemic disturbances" [2–5]. A consequence of trauma is polytraumatism, which is classified according to the Trauma Committee of the American College of Surgeons—ATLS (*Advanced Trauma Life Support*), as being "in two or more organ systems, being necessary that a combination of these lesions represent a vital risk to the patient" [5].

In Brazil, this scenario is likely to be repeated, due to the uncontrolled growth of cities, as well as the marginalization of its population, and disparities in social conditions and lifestyles, which characterize it as a conflict-ridden society with a large amount of violence and traffic accidents. This represents a public health problem, with trauma being a relevant consequence [3,6].

In view of this worldwide scenario, the health institutions and their professionals have as a venue for treating these events the intensive care units (ICUs), which are intended for the treatment of recoverable patients at risk of death who need continuous and specialized care [1,7,8]. The need for ICU hospitalization is multifactorial. ICUs are intended for cases of medium and high complexity. Intensive care is aimed at assisting the individual in a severe state in an integral manner, with human resources, materials, and high technology equipment available [9–11]. Treatment and hospitalization in ICUs have contributed to greater survival of patients with the most diverse diseases, due to the development of hard, light-hard, and light technologies. In addition, the need to keep up with daily personal and work activities imposes on the daily lives of citizens a greater health risk and a greater number of long-term complications due to exposure to stressors, among them an increase in the number of circulating vehicles, culminating with higher rates of health damage mainly from accidents involving automotive vehicles. This leads to increasingly complex services and raises costs [8,11].

Treatment costs (including rehabilitation and accident investigation), as well as reduction or loss of productivity, make this process long and costly for health institutions and society as a whole. In addition, studies show that trauma victims are usually in the age range of 5–44 years, most of them men who are in their most productive phase, including economically [1,2]. According to the American Trauma Committee, the estimate for 2020 is that one in ten of these individuals will die from trauma [6].

In ICUs, the control of these patients is done through physical examination performed by the multiprofessional team that works in this environment and image and laboratory examination. The laboratory procedures most used for diagnostic purposes involve analysis of the chemical and cellular constituents of the blood. The most requested blood elements are sodium, potassium, calcium, phosphorus, magnesium, serum creatinine and urea, prothrombin time (PT), activated partial thromboplastin time (APTT), lactic acid, arterial blood gas, capillary glycemia, fasting glucose, blood count, and platelets. However, other biological constituents are also used for these purposes, such as urine, cerebrospinal fluid, feces, and saliva [12,13].

Conventional methods for blood serum analysis involve invasive procedures, and these cause pain and may present health risks, due either to the imperfection of the collector or the poor health condition of the individual in addition to the high cost [14–16]. Given this context, studies have verified the use of saliva for the clinical control of patients with systemic or localized diseases in the mouth, since it can be easily collected non-invasively when compared to blood collection [17,18].

According to Kaufman et al., (2002) [17] the use of saliva for diagnostic tests in patients with systemic diseases has been used for offering distinct advantages over blood. It can be collected non-invasively by individuals with technical training. In addition, saliva can provide a cost-effective approach for tracking large populations. Total saliva (as in the evaluated article), however, is most often used for the diagnosis of systemic diseases, as it contains serum constituents. Therefore, the authors point out that saliva analysis can be useful for the diagnosis of hereditary and autoimmune diseases, malignant and infectious diseases, and endocrine disorders, as well as for the assessment of therapeutic drug levels and the monitoring of illicit drug use.

Saliva is a biological fluid secreted continuously by salivary glands. Its inorganic part is composed of ions and mineral salts such as chloride, bicarbonate, chlorine, phosphate, iodide, bromide, fluoride, sodium, potassium, and calcium, and the organic part is made up of proteins and enzymes. Its main function is the protection of the buccal tissues as well as other physico-chemical and biochemical properties that aid in speech, food processing and dental protection, and remineralization [17–19]. Studies indicate that the composition of saliva reflects the tissue levels of therapeutic drugs, hormones, and immunological molecules [17]. Studies have revealed the effectiveness of salivary analysis as a substitute for serum analysis in patients with diabetes mellitus (DM) [20] and chronic renal disease (CKD) [21], in children and adolescents [22], and in patients with acute traumatic brain injury ICU [23]. In these individuals hospitalized in an ICU, an increase in cortisol and salivary amylase was observed in children and adolescents (surgical, trauma, oncology), and these results were correlated with increased severity [22]. In adults, there was an increase in cortisol [23]. As far as we know, these are the only studies performed with patients hospitalized in ICU.

In the oral cavity, saliva from the ducts mixes with other secreted products present there, such as oral epithelial desquamative cells, nasal cavity mucus, food debris, microorganisms, organic and inorganic compounds, bacterial metabolism products, mucosal transudate and exudate of gingival grooves; the mix is then called total saliva [17,18]. Salivary flow directly affects the composition of saliva and can be obtained in two ways: unstimulated saliva and stimulated saliva. Unstimulated saliva may correspond to half of the saliva flow stimulated in non-complex patients, and the amount of saliva available is directly linked to oral homeostasis [16,18,19].

When salivary markers (urea, creatinine, glucose, and calcium) were first evaluated in polytrauma individuals in the ICU, the use of total saliva was chosen. Previous studies have shown that saliva has reliable biological properties for analysis of the composition of the oral microbiota, even when there are alterations in the salivary flow, which can be caused by variations in the circadian cycle and factors such as stress and exercises and other systemic alterations, situations present in the ICU [20,22,23,24].

In addition to the serum parameters used to evaluate critical individuals, other tools have been used to subsidize and predict the prognosis of these individuals, such as the Acute Physiology and Chronic Health Evaluation *(APACHE) [25–27]*. This index is a point-based assessment system that emerged from the need to classify groups of patients admitted to ICUs. APACHE is based on the severity of the disease and the estimation of the risk of death through standardized information [26,27]. It was based on the hypothesis that the severity of the acute disease can be quantified by the degree of abnormality of physiological variables. Age and the presence of chronic diseases prior to admission to the ICU were also considered to decrease the physiological reserve and, therefore, directly reflect the patient's survival [26,27]. These variables, considered influential for patient survival, were selected for APACHE [27].

The APACHE system was developed, modified, and validated over three decades of studies: APACHE [25], APACHE II [26], APACHE III [27] and APACHE IV [28]. Among all APACHE systems, APACHE II has been widely used in Brazil and worldwide [28,29]. The Brazilian Ministry of Health, in its ruling number 3.432 of 2010, considered the existence of several indices but recommended that APACHE II be used in all ICUs because it is consecrated by use [28]. In 2010, with the publication of the Resolution of the Collegiate Board of Directors (RDC) No. 7, it recommended that IP APACHE II be used in all ICUs because it is a system of disease severity classification recommended in the specialized scientific literature [30].

**Fig 1:**
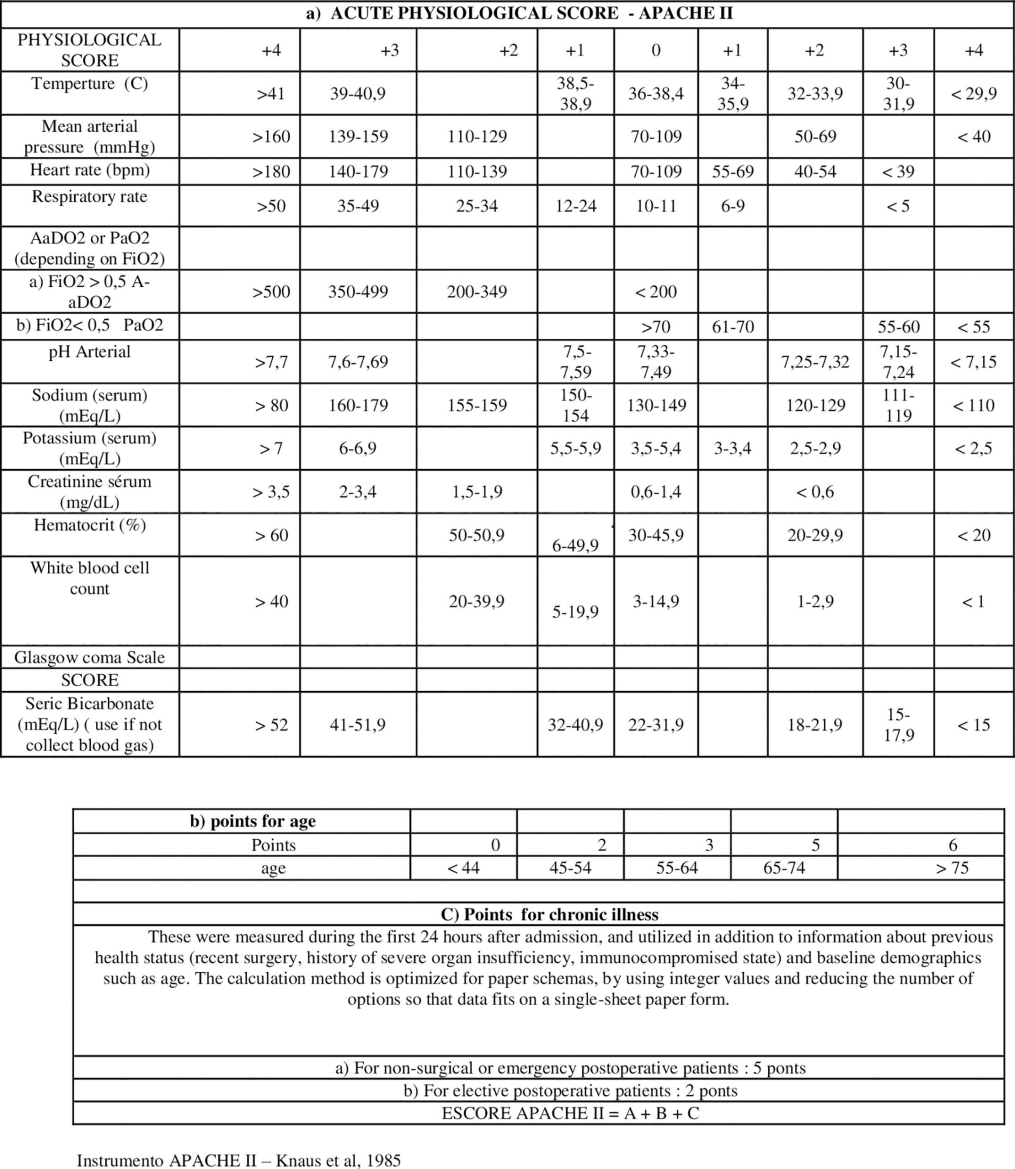
Lethality rate predicted by APACHE II. Source: Knaus WA, Draper EA, Wagner DP, Zimmerman JE. (1985).

IP—APACHE II (Fig 1) is considered easy to apply. The clinical and laboratory variables it uses are routinely collected during intensive care and validated for a wide range of diagnoses [25,26,30,31].

### Goals

Due to the lack of studies, to our knowledge, that evaluate the salivary parameters in polytraumatized patients in the general ICU, the possibility of salivary parameters being potential indicators of clinical prognosis, along with blood, the ease and noninvasive way of obtaining saliva and the possibility of doing so, and the possible correlation between serum and salivary parameters, this study is justified.

In view of the above, our objectives were:

- To characterize the sociodemographic profile and type of lesions in polytraumatized patients hospitalized in the ICUs of a university hospital in the city of Curitiba, Paraná;
- To compare blood serum levels of pH, sodium, potassium, urea, creatinine, glycemia, and calcium with parameters of normality in polytraumatized patients hospitalized in ICU, stratified according to APACHE II, from 0 to 19 and greater than 20;
- To verify if there were variations in these levels between the periods of 0, 48, and 72 hours of ICU stay;
- To compare the salivary levels of urea, creatinine, glucose, and calcium with parameters of normality in polytraumatized patients hospitalized in ICUs, stratified according to APACHE II, from 0 to 19 and greater than 20;
- To verify if there were variations in these levels between the periods of 0, 48 and 72 hours of ICU stay; and
- To correlate the salivary and blood parameters of these patients.

## Materials and methods

### Study design

This was a prospective observational study of a quantitative nature, in which the same patients were evaluated at admission, 48 hours, and 72 hours.

### Ethical procedures

Ethical and legal aspects were considered, since the study was conducted in accordance with Resolution 466/12 of the National Health Commission. The project was approved by the Ethics and Research Committee of the Pontifical Catholic University of Paraná (1,358,439; CAAE: 51055515.3.0000.0020). The research was authorized by the local Research Ethics Committee (no. 1.358.439), the Technical Department of Cajuru University Hospital (HUC), PR, and the Intensive Care Service of the HUC, PR (S1, S2 and S3 Text).

### Location of the study

The study was conducted in the ICUs of a university hospital administered by the Paranaense Association of Culture. Currently, the hospital is part of the Marista Group Health Area. It is located in the city of Curitiba, Paraná. These units were chosen because they are among the largest urgency and emergency hospitals in the state and, since 2006, have been accredited by the Ministry of Health as a high complexity unit in orthopedics, traumatology and renal transplants.

### Sample selection

The study sample consisted of seventy polytraumatized patients admitted to the hospital who needed hospitalization in the ICU from April 2016 to October 2017. During that period, 857 patients were consecutively admitted to the ICUs. Of these, 180 patients were included in the inclusion criteria because they had available test results.

The inclusion criteria were: between 15 and 65 years of age, polytrauma patients hospitalized at ICUs, with a period of hospitalization longer than 24 hours and up to 72 hours (when the last collection of blood and saliva was performed), intubated with oxygen therapy support, Glasgow Coma Scale (GCS) less than or equal to eight (< or = 8), or Ramsey Scale 5 or 6, regardless of gender. 180 patients met the inclusion criteria.

As exclusion criteria: septic patients at the time of hospitalization, rehospitalization, individuals in brain death at admission and during the collection period, inability to obtain the consent term from their legal representative, unidentified individuals, clinical individuals, known prior co-morbidities, pregnant women, and individuals presenting active bleeding of oral mucosa as well as those with suspected oral infection or known oral infection and insufficient amount of saliva collected. In this way, 28 patients had charts with incomplete data; 5 evolved to encephalic death within 48 hours of hospitalization; 5 family members did not agree to participate in the research; 23 clinical patients were admitted; 22 patients came from the internment ward; 18 patients had sepsis; 9 presented with severe bleeding in the oral cavity.

### Data collection

Data referring to the general characterization of the patient were collected through a structured interview and from the medical records. Due to non-authorization by the (unconscious) patient, the researcher approached direct relatives or those responsible for hospitalization at the time of admission to request permission to collect data. Those who agreed to take part in the study signed an Informed Consent Form (S4 Text). The IP APACHE II variables were collected according to the original proposal (S5, S6, S7 and S8 Text) (APACHE II groups), on a form filled out by the ICU medical team 24 hours after the patient's hospitalization. The data collected were the following: sex, age, marital status, schooling, remunerated activity, type of accident, accident period, trauma types, and blood transfusion (S9 Text). To perform the predictive gravity analysis of the 70 patients, we adapted the original 1985 Knaus Scale, for only the two following classes: I: APACHE II: 0 to 19 and II: APACHE II: 20 to 71 [25,26].

### Serum collection and analysis

Serum blood results were collected from medical records. The blood collection method followed the routine collection of blood from the ICUs, performed by the nurses from radial and/or femoral artery puncture, shortly after receiving the patient in the ICU. The other samples (48 and 72 hours) were collected directly using the PAM device (in the radial and femoral artery), between 4 and 6 o'clock in the morning, according to the laboratory routine of the service. A mean of ten to fifteen mL were withdrawn in a syringe and were then deposited in disposable gel vacuum tubes (BD-Becton Driver, Franklin Lakes, New Jersey) without anticoagulant. The biochemical analysis of the blood was carried out in the Cajuru-HUC laboratory of clinical analysis (Curitiba, PR, Brazil), and the levels of sodium, potassium, urea, creatinine, calcium, and glucose were quantified using the automatic biochemistry system Integra 400 (Roche, Japan). The pH value was automatically quantified by the Cobas b121 blood gas analyzer (Roche, Switzerland).

### Salivary collection and analysis

The total saliva samples were collected by the researcher (MHMC); the sample was calibrated by a specialist (STL) and obtained at 3 different moments: at admission, and at 48 and 72 hours after admission. The procedure was performed by positioning a suction probe 14 or 16, adapted to an airway secretion collecting bottle (bronchus, CAMAHE, Curitiba, Paraná), to a mechanical aspirator. The tip of the catheter was moved in the lingual floor and lingual face of the premolars, being moved from the premolar on one side to the other and passing through the lingual floor in the region of premolars, canines, and central and lateral incisors. The movement was repeated for five uninterrupted minutes. The collected saliva was stored in an airway secretion-collecting vial that was attached to the aspirator. After the collection of saliva, the bottles were hermetically sealed, placed in a thermal container, and sent for freezing at (-) 60°C (Consul 415 freezer, Curitiba, PR) to the biochemistry laboratory, located at the School of Life Sciences of PUC PR. Biochemical analyses of saliva were performed by an analyst (MHMC), with colorimetric tests from Labtest Diagnostic (Labtest, Lagoa Santa, Minas Gerais). Urea, creatinine, glucose, and calcium were tested. Values were expressed as mg/dL.

For the standardization of the collection technique, we performed a pilot study on five patients.

### Statistical analysis

The data were analyzed in a database developed in the IBM STATISTICS SPSS 25.0 program, (SPSS Inc, Chicago, Illinois). An exploratory descriptive analysis of the variables (median, standard deviation, 95% confidence interval for the mean, minimum, and maximum) was performed for each of the categories of the APACHE II variable, in order to compare the findings with the variation range of the gold standard, as described in the literature.

### Serum analysis

The Kolmogorov-Smirnov normality test was performed for serum pH, sodium, potassium, urea, creatinine, glucose, and calcium at admission, 48 hours, and 72 hours, according to each category of independent variables considered (Apache II, TBI, facial trauma, spine trauma, chest trauma, abdomen trauma, trauma of an extremity). Since the dependent variables did not present a normal distribution in at least one of the categories of the independent variables, the comparison between the independent variables for two categories at a given time was made using the non-parametric Mann-Whitney U test. The comparison between the times (Admission x 48, Admission x 72, and 48 x 72) was made using the non-parametric Wilcoxon test for paired samples for each category of the APACHE II independent variable. For the verification of the correlations of two non-parametric variables, the Spearman test was used, with 0.00–0.30 considered weak; 0.30–0.60 moderate, 0.60–0.90 strong, and 0.90–1.00 very strong. The level of significance adopted in all tests was 5%.

### Salivary analysis

The Kolmogorov-Smirnov normality test was performed for the calcium variable at admission, calcium 48 h and calcium 72 h. The sample size was 25 in the pairing of calcium at admission x 48 h calcium, and n = 24 in the pairing of calcium 48 h x calcium 72 hours. The test showed that the calcium at admission variable was not normal; the opposite was observed for 48 h calcium and 72 h calcium. Thus, the option was made to choose the non-parametric Wilcoxon test for paired samples to compare calcium at admission x calcium 48 h, and Student's t test for paired samples to compare calcium 48 h x calcium 72 h. For the other variables, since the dependent variables did not present a normal distribution in at least one of the categories of the independent variables, the comparison within each time between the independent variables for two categories was made using the non-parametric Mann-Whitney U test.

For each category of the APACHE II independent variable, the comparison between times (admission x 48, admission x 72, and 48 x 72) was made using the non-parametric Wilcoxon test for paired samples. For the verification of the correlations of the two non-parametric variables, the Spearman test was used, considering 0.00–0.30 weak, 0.30–0.60 moderate, 0.60–0.90 strong, and 0.90–1.00 very strong. The level of significance adopted in all tests was 5%.

## Results

Of the patients in this study, 61 (87%) were males and 9 (13%) were females. The mean age was 27 years (39%). Regarding marital status, 28 (40%) were unmarried, 38 (54%) were married, and 3 (4%) were widowed. In relation to schooling, 39 (56%) had incomplete high school. As for reported monthly income, 64 (91%) were engaged in a paid activity. 26 (41%) received values ranging from less than or equal to one minimum wage, and 38 (59%) from two to four times the minimum wage (Table 1).

**Table 1:** Distribution of polytrauma patients admitted to the ICUs of a university hospital, with regard to sociodemographic data.

Sociodemographic variables	Nº	59%
**Sex**		
Male	61	87%
Female	9	13%
**Age group (years)**		
15–29	27	39%
30–50	35	50%
51 e +	8	11%
**Marital status**		
Not married	28	40%
Married	39	56%
Widowed	3	4%
**Schooling**		
Incomplete fundamental	10	14%
Complete Fundamental	12	17%
Incomplete Secondary	39	56%
Complete Secondary	9	13%
**Paid activity**		
No	6	9%
Yes	64	91%
**Remuneration (x the minimum wage)**		
< or = 1	26	41%
2 to 4	38	59%
5 or +	-	-

Data collected in Curitiba, Paraná, Brazil, 2017–2018.

Regarding the type of trauma, blunt trauma was present in 56 (80%) and penetrating trauma in 14 (20%) patients. Of the blunt traumas (contusions), collisions involving motor vehicles account for 37 cases (53%), 28 (40%) of which involving cars and 9 (13%), motorcycles. Among the penetrating traumas, firearm injuries and white-weapon injuries (Table 2) stood out.

**Table 2:** Distribution of patients according to the type of accident that caused the trauma.

Type of accident	nº	59%
**Contusive Trauma**		
Collision	28	40
Aggression	9	13
Running over	9	13
Falls	9	13
Electric shock	1	1
**Penetrating trauma**		
Firearm	9	13
White-weapon	5	7

Data collected in Curitiba, Paraná, Brazil, 2017–2018.

In comparison with the normality parameter, a decrease was observed for: pH at admission in both classes; creatinine on admission to Class I and at 48 and 72 hours for both classes; K^+^ in 48 hours in Class II; on admission in both classes and in 72 hours in Class I. An increase in the value of the parameters: urea 72 hours in Class II, glucose at all times and classes, calcium 48 h for both classes.

Comparing the classes, Class II individuals presented higher values of Na^+^ at 48 and 72 hours when compared with Class I subjects.

Comparing the times among the Class I subjects, there was a reduction in creatinine at times 48 and 72 hours in relation to admission and an increase in calcium 48 hours when compared to admission.

Comparing the times in the Class II subjects, pH and Na^+^ increased at 48 and 72 hours in relation to admission. K^+^ decreased between admission and 48 hours and increased between 48 and 72 hours. Urea increased from 48 to 72 hours. Creatinine decreased from admission to 48 and 72 hours. Calcium increased from admission to 48 hours and decreased from 48 to 72 hours (Table 3).

**Table 3:** Results of APACHE II and serum markers (PH, Potassium, Sodium, Urea, Creatinine, Glucose and Calcium) of 70 polytraumatized individuals in three times after admission to HUC ICUs, Curitiba, Paraná.

Variable	APACHE II	N	Medium	95% confidence interval for mean		APACHE II	N	Medium	95% confidence interval for mean		Golden pattern
				Lower limit	Upper limit				Lower limit	Upper limit	
CLASS I						CLASS II					
**Number of lesions**	**0–19**	**11**	2.00A	1.90	3.00	**20–71**	**59**	2.00	2.13	2.60	NA
**pH—Admission**			7.30Aa	7.31	7.40			7.36Aa	7.32	7.37	**7.35–7.45**
**pH—48 hours**			7.41Aa	7.38	7.43			7.42Ab	7.39	7.42	
**pH—72 hours**			7.40Aa	7.37	7.44			7.42Ab	7.37	7.42	
**K** ^**+**^** - Admission**			3.90Aa	3.63	4.67			4.10Aa	3.89	4.25	**3.5–5.1 mEq/L**
**K**^**+**^ **- 48 hours**			3.90Aa	3.60	3.94			3.50Ab	3.44	3.76	
**K**^**+**^ **- 72 hours**			3.90Aa	3.66	4.05			3.70Ac	3.63	4.04	
**Na**^**+**^** - Admission**			139.00Aa	138.27	141.61			139.00Aa	138.71	141.30	**136 – 145mEq/L**
**Na+ - 48 hours**			137.00Aa	135.81	140.73			142.00Bb	141.16	143.75	
**Na+ - 72 hours**			137.00Aa	135.75	140.07			143.00Bb	142.12	145.28	
**Urea Admission**			33.00Aa	22.96	38.30			33.00Aa	31.35	51.09	**16.6–48.5mg/dL**
**Urea—48 hours**			24.00Aa	19.94	36.77			32.00Aab	33.80	48.43	
**Urea—76 hours**			33.00Aa	24.47	41.16			40.00Aac	40.94	59.18	
**Creatinine—Admission**			0.62Aa	0.50	1.02			0.70Aa	0.71	0.96	**0.70–1.20 mg/dL**
**Creatinine—48 hours**			0.51Ab	0.42	0.73			0.45Ab	0.46	0.89	
**Creatinine—72 hours**			0.51Ab	0.42	0.75			0.48Ab	0.45	1.01	
**Glucose—Admission**			151.00Aa	119.44	169.46			148.00Aa	146.45	172.42	**70–115 mg/dL**
**Glucose—48 hours**			143.00Aa	121.94	169.32			1444.00Aa	139.65	167.53	
**Glucose—72 hours**			144.00Aa	112.80	157.19			148.00Aa	139.20	156.85	
**Calcium—Admission**			1.15Aa	1.05	1.32			1.11Aa	1.03	1.38	**1.16–1.32 mg/dL**
**Calcium—48 hours**			1.70Ab	1.25	1.67			1.45Ab	1.36	1.52	
**Calcium—72 hours**			1.17Aab	1.09	1.31			1.16Ac	1.15	1.24	

pH = potential Hydrogen ion; Na^+^ = sodium; K^+^ = potassium.

A-z = APACHE II: 0–19; B = APACHE II: 20 to 71.

Mann-Whitney test = Distinct capital letters indicate differences between classes.

Wilcoxon Test = Distinct lowercase letter reveals differences between times in each class.

NA = does not apply.

Regarding normality, all parameters evaluated increased in both classes and in the three times. There was an increase in urea at admission, glucose at 72 h, and calcium at 48 h in Class II individuals, compared to Class I. Among Class I subjects, urea showed an increase between admission and 48 hours, and calcium decreased between admission and 48 hours. As for Class II, there was a decrease in urea between 48 and 72 hours (Table 4).

**Table 4:** Salivary levels of urea, creatinine, glucose, and calcium at admission, 48 h and 72 h, in Classes I and II of APACHE II, and normality standard.

Variable	APACHE II	N	Median mg / dL	95% confidence interval for mean		APACHE II	N	Median mg / Dl	95% confidence interval for mean		Gold standard
				Lower limit	Upper limit				Lower limit	Upper limit	
CLASS I						CLASS II					
**Urea—Admission** *	**0–19**	**11**	24.00Aa	16.41	36.86	**20–71**	**59**	53.00Ba	57.14	91.73	**20 mg/dL**
Urea—48 hours*			49.00Ab	19.05	118.15			54.00Aab	69.87	110.76	
**Urea—72 hours** *			31.00Aab	17.09	77.91			40.00Aac	48.01	79.20	
Creatinine—admission *			0.72Aa	0.54	1.15			0.69Aa	0.75	1.00	**0.12–0.16 mg/dL**
**Creatinine—48 hours** *			0.78Aa	0.66	0.93			0.56Aa	0.60	1.21	
**Creatinine—72 hours** *			0.62Aa	0.47	0.96			0.59Aa	0.67	1.23	
**Glucose Admission** *			103.21Aa	28.98	17.30			109.46Aa	71.27	124.64	**5.6–18.4 mg/dL**
**Glucose 48 hours** *			111.64Aa	46.75	196.23			108.52Aa	78.08	132.15	
**Glucose 72 hours** *			21.59Aa	-116.99	56.68			103.13Ba	72.46	125.91	
**Calcium—Admission** *			49.35Aa	29.41	53.66			33.45Aa	28.73	38.46	**5–7 mg/dL**
**Calcium—48 hours** **			12.61Ab	6.23	33.73			32.07Ba	26.77	37.03	
**Calcium—72 hours** **			17.43Aab	12.68	43.62			30.69Aa	26.07	34.76	

UTIs of HUC Curitiba, Paraná. A = APACHE II: 0–19; B = APACHE II: 20 to 71.

Mann-Whitney test = Distinct capital letters indicate differences between classes.

*Wilcoxon test

** Student’s t test = Distinct lowercase letter reveals differences between times in each class.

There was a positive correlation between serum and salivary parameters: in creatinine, very strong at admission and strong at 48 and 72 hours, and in regular calcium in 72 hours (Table 5).

**Table 5:** Spearman's correlation coefficient between serum and salivary parameters.

Spearman Correlation Coefficient	Saliva Urea on Admission	Saliva Urea 48 hours	Saliva Urea 72 hours	Saliva Creatinine in Admission	Saliva Creatinine 48 hours	Saliva Creatinine 72 hours	Saliva Glucose on Admission	Saliva Glucose 48 hours	Saliva Glucose 72 hours	Saliva Calcium on Admission	Saliva Calcium 48 hours	Saliva Calcium 72 hours
**Blood Urea on Admission**	0.065											
**Blood Urea 48 hours**		0.103										
**Blood Urea 72 hours**			- 0,025									
**Blood Creatinine on Admission**				.954*								
**Blood Creatinine 48 hours**					.735*							
**Blood Creatinine 72 hours**						.745*						
**Blood Glucose on Admission**							0.025					
**Blood Glucose 48 hours**								0.109				
**Blood Glucose 72 hours**									0.196			
**Blood Calcium on Admission**										- 0,058		
**Calcium Blood 48 hours**											0.013	
**Calcium Blood 72 hours**												-,303*

* P <0.05 (2 extremities). ICUs of HUC Curitiba, Paraná.

## Discussion

This is the first prospective observational study that has investigated the epidemiological profile, serum, and salivary levels in polytrauma patients hospitalized in ICU, to have observed a positive association between the severity of the patient’s condition and variation in these metabolites.

The patients’ profile was similar to that found in studies with polytraumatized patients in the ICU, with a majority of male patients, young individuals, inserted in the labor market and economically active [1–3]. One hypothesis for this profile would be that adolescents and young adults are more exposed to accidents and other violence, mainly due to inexperience, search for emotions, pleasure in experiencing sensations of risk, impulsivity, and abuse of alcohol or drugs [32]. Regarding the types of accidents, the highest percentage was found to be victims of traffic accidents, which are mainly responsible for the injuries, corroborating the findings of previous studies [33,34]. It is known that over the past 10 years, more than one million people have been disabled due to mechanical traumas in the world, with traffic accidents being mainly responsible for these rates [33–35]. It is worth noting that hospitalization for trauma resulting from a traffic accident may correspond to more than 40% of all ICU hospitalizations, depending on the hospital [7,9]. This represents high hospital costs, material losses, social security costs, and great suffering for the victims and their relatives [36–38].

Regarding the serum parameters evaluated, it was noted that blood acts as a buffer solution, which prevents its pH from undergoing major changes. The pH of blood and extracellular fluids remains in the range of 7.35 to 7.45 [39–41]. The organism supports pH changes ranging from 6.8 to 7.8. Decreased blood pH is called acidemia or acidosis; increased pH of blood, alkalemia or alkalosis [40]. Polytraumatic situations culminate in functional and physiological alterations, mainly due to the involvement of one or more vital systems [5,39], as observed among the individuals of this study. If this ratio is altered in any way, it can cause serious damage to the body with profound metabolic changes, which can cause death [5,39,40]. Patients with polytraumas tend to present alterations in the respiratory pattern (either by direct trauma in the thoracic region or by stress), and with this, the amount of HCO3- (aq) increases greatly in relation to H2CO3 (aq); thus, the pH of the blood rises, triggering an alkaline disturbance. This is due to a very rapid breathing, which decreases the amount of CO2 in the body, shifting the chemical balance to the left and decreasing the amount of H+ (aq) (hence the pH increases) [39]. In addition to polytraumatism, other factors also trigger hyperventilation and alkalosis in individuals, such as: drug use, hyperthermia, excessive exercise, cirrhosis, aspirin overdose, and lung diseases [10,40,41].

Severe acid-base balance alterations are potentially critical, especially when they develop rapidly (a feature very similar to those found in the study population), because they are "polytraumatized". Such abnormalities may directly cause several organic dysfunctions, such as cerebral edema, fractures, decreased myocardial contractility, pulmonary vasoconstriction, and systemic vasodilation, among others [42,43]. Acidosis and alkalosis (respiratory, metabolic, or mixed) are common and clinically significant phenomena in poly-trauma patients in the ICU [42–44].

In this study, the pH value underwent a left shift in the two APACHE II classes at admission. This is justified mainly by the individual having suffered a trauma and by the degree of stress by means of gravity, characteristic in this study. After 48 and 72 hours, a normalization of this element occurred, and the possible reason for this is that all patients were on mechanical ventilation (MV) from admission and treatments instituted in the ICU, thus maintaining their progressive control of the initial condition [40–42]. MV is a therapy applied in several clinical situations, depending on the severity and risk of death of patients suffering from polytrauma, in order to adequately maintain the levels of O2 (oxygen) and CO2 (carbon dioxide) gases, indispensable for the maintenance of vital organs. Increasingly, the ICU team uses these technologies with different resources for the control and analysis of respiratory parameters supplied by the ventilator, which will guide the teams to the clinical decisions that these patients undergo [42].

To maintain pH within limits compatible with vital processes, the body has a series of regulatory mechanisms, which are: buffer system (instantaneous), between the respiratory (minutes) and the renal (hours to days) [40,41]. It is emphasized that pH variation leads to frequent and clinically significant disorders in severe ICU patients regardless of etiology (polytrauma, sepsis, or shock) [39–41]. Previous studies have shown that polytrauma patients have a higher risk of developing severe metabolic abnormalities, especially acute metabolic acidosis, which is associated with an increase of 0.6 mEq/L in the serum potassium concentration for each 0.1 decline in pH [39,40,43].

Sodium and potassium electrolytes are important for maintaining this equilibrium. In APACHE Class II individuals, K^+^ was normal at admission, decreased at 48 hours compared to the gold standard, and returned to normal at 72 hours. Class II subjects presented with higher values of Na^+^ at 48 and 72 hours when compared to Class I individuals but within the parameters of normality. Although the results obtained show little variation in relation to normality, it is worth mentioning that sodium and potassium are essential components of body fluids, such as blood, saliva, and urine, helping to regulate the distribution of water throughout the body and playing a fundamental role in basic acid balance. Each and every discomfort to the body, characteristic of polytraumatized individuals, affecting target organs such as the kidneys, heart, and liver, culminates in a risk to the regulation of the volume and composition of body fluids as well as the maintenance of electrolyte balance [39,43,44].

In addition to the above, it is worth mentioning that variation in serum sodium concentration is an important determinant of blood osmolarity, since hyponatremia and hypernatremia are associated with severe brain disorders (cerebral edema), a situation that is very possible in polytraumatized patients [44]. The possible variations between Na^+^ and K^+^ reinforce the fact that in these polytraumatized individuals there is greater difficulty in maintaining normal osmolality because they tend to retain Na^+^ and water in the extracellular medium and increase the concentration of K^+^ in the intracellular medium over time, due to the long period of sedation and MV, absence of calorie intake necessary for the maintenance of life, great volume losses, hemodynamic instability, and risk of infections [40–43].

Urea and creatinine are two substances present in blood and saliva which, when dosed, make evaluation of renal function possible [43,45]. In this study, elevated serum urea levels above the 72-hour normality parameters in Class II subjects and a decrease in creatinine at admission in Class I and at 48 and 72 hours in subjects in both classes were observed.

In view of the findings, it is pointed out that for proper renal functioning, it is necessary to evaluate four physiological functions: blood flow, glomerular filtration, tubular function, and permeability of the urinary tracts. One or more of the factors mentioned above are altered in polytraumatized individuals. The inadequate functioning of the kidneys predisposes an inability for glomerular filtration, causing an increase in urea and creatinine concentrations as a consequence of the increased protein catabolism present in polytraumatized individuals. However, when serum levels, such as creatinine, are decreased, this also indicates severe changes in renal perfusion, which are usually caused by a decrease in renal blood flow and/or severe dehydration, excessive fluid replacement [40,41,46], situations experienced by individuals in both classes in this study.

Polytrauma patients have a rate ranging from 2% to 5% of risk of developing alterations in renal function, regardless of underlying trauma, due to the great influence of factors such as: hypovolemia, septic shock, aminoglycoside use, and contrast imaging tests, which usually progress to pre-renal and renal disorders [43,44,47,48]. Among the pre-renal causes, uremia related to an increase in protein catabolism and stress due to trauma, such as that observed in this study in relation to urea values 72 hours after admission in Class II individuals, stands out. Among the renal causes, acute tubular necrosis, usually caused by renal hypoperfusion and/or endogenous and exogenous nephrotoxins, are the most common factors during the care of critically ill ICU patients [40,42,43,44,46]. Information on renal function in these individuals in the ICU, through the control of electrolytes such as urea and creatinine, may contribute to the verification of situations that would lead to multiple organ failure during the hospitalization period, culminating in evolution and negative outcomes in relation to the prognosis [40,41,49]. Polytraumatized individuals of both classes I and II presented elevated levels of urea and salivary creatinine when compared to normality. Due to the lack of studies that perform this evaluation in polytrauma patients, normality for these two markers was drawn from values presented in previous studies performed with patients with chronic renal disease (CKD) on hemodialysis treatment and a control group that did not present with CKD [17–19]. Urea and creatinine are two substances present in the salivary flow and in the bloodstream that are dosed when there is a need to evaluate renal function in specific population groups (CKD and DM) [17,18,39]. Failure of renal function can occur due to the quality and intensity of aggressive stimuli to the kidneys, which causes loss of the functional unit of this organ, a scenario that is quite possible in polytraumatized patients in the ICU [39,40]. Salivary fluid has potential in other groups, since it is considered an excellent material for systemic verification and oral disease, among others [17,18,19,50].

The polytraumatized patients in this sample do not have CRF but may develop acute renal failure (ARF), which is a syndrome characterized by abrupt and persistent deterioration of renal function, resulting in the inability of the kidneys to excrete nitrogenous slags and to maintain hydroelectrolytic homeostasis [39]. Associated with this are the interventions used for resuscitation, such as the use of drugs that alter or make difficult glomerular filtration; and thus, the release of toxins in the body is a consequence [25,27, 51].

In view of the above, we stress that in our study salivary creatinine showed a very strong correlation in relation to the blood when dosed at admission; and a strong correlation in 48 h and 72 h, thus being a marker as effective as blood in acute situations and also having the advantage of early detection of changes.

Comparing the salivary urea of nephropathic individuals with normal individuals, it was identified in the first hour of fasting that the two samples presented a very high value when compared to blood urea [52]. Bearing in mind the investigation and the need for greater scientific confirmation regarding a special group of individuals such as those in this study, our findings are consistent with previous reports, even though in other categories we infer that it is important to highlight the possibility of using salivary creatinine and urea analysis for diagnosis and evaluation, not only of chronic kidney disease but also of acute renal disease, especially in polytrauma patients [18,19,52,53].

Serum glucose presented significant changes at the three times and in the two APACHE II classes, with values increasing in relation to the gold standard. Hyperglycemia is common in critical individuals and is attributed to the physiological response to trauma, due to the high degree of stress, represented by increased cortisol [54]. Glycemic levels are maintained physiologically by the interaction between insulin secretion, cellular uptake of glucose (glycolysis and glucogeneogenesis), hepatic glucose production (glycogenolysis and gluconeogenesis), and intestinal absorption [55–57]. Increased glycemia corresponds to increased metabolic demands in these individuals, and in most cases is accompanied by hyperinsulinemia and endogenous increase of liver production and also by causes such as increased glycemia in enteral and parenteral diets, dialytic solutions, and glucocorticoid use; vasopressor substances are routinely used in this study population [58–62].

According to the *American Disease Association* (ADA) and *Brazilian Society of Diabetes* (BDS), the reference value of fasting serum blood glucose obtained by puncture is less than 100 mg/dL for diabetic patients, and 100 to 125 mg/dL for diabetic patients with readings greater than or equal to 126 mg/dL (*National Diabetes Data Group*) [62–64]. Another common practice in the glycemic evaluation of patients in the ICU is a capillary check at the bedside, which can present the following results: lower than 70 mg/dL: hypoglycemia; 70 mg/dL to 140 mg/dL: normal; 140 mg/dL to 200 mg/dL: pre-diabetes; greater than 200 mg/dL: diabetes. These values have relevance, as long as they are obtained in the proper way with regard to the calibration technique [62–67]. The values considered here were 70 to 115 mg/dL [62]; the values of salivary glucose considered normal were: 5.6 to 18.4 mg/dL [68].

The glucose in the saliva presented an increase in the three times and in the two classes, with respect to the standard of normality. Studies indicate that saliva in type 2 diabetic individuals has potential for the detection of oral diseases as well as in the control of type 2 diabetes in follow-up [53]. Along these lines, both blood glucose and salivary glucose values converged, showing an increase in both. However, in the present study we did not verify correlation between serum and salivary glucose. A previous study [44] showed that in diabetic patients, the concentration of salivary glucose was much higher than in the control group.

Serum glycemic monitoring and its effects have been a concern in studies in the last decade. The verification of blood glucose and the mortality rates of critically ill patients in the ICU appear to have disparities in relation to three aspects: insulin administration, frequency of sample collection, and target amplitude of glycemic indexes [62,64,65]. Serum corrections for hyperglycemia were performed through blood and capillary serum evaluation. This fact may have interfered in the findings of this study, implying that these individuals could present a higher value of serum glucose in relation to what was found. Although this is one of the limitations of this study, it is emphasized that this intervention was performed in all individuals [66–68].

The serum calcium evaluated in this research is part of the group of essential mineral elements and needs to be acquired, mainly through food. The values of salivary calcium discussed here refer to the following readings: 5 to 7 mg/dL; however, these values are for healthy individuals, with the collection taken at rest–and they vary widely among individuals. As previously mentioned, this was the first study to evaluate the salivary index of polytraumatized patients in intensive care units (ICU), so there are no reference values to be compared [69].

The highest concentration of calcium in the human body occurs in the bone matrix (99%), distributed between bone and teeth in the form of calcium phosphate. The remaining calcium portion (1%) is located in the intra- and extracellular medium, mainly associated with the protein carrier, such as albumin. In polytraumatized individuals, bone lesions usually culminate in fractures and blood loss, compromising the production and balance of this element, which is vital to the proper functioning of the organism. There are several important functions attributed to calcium, mainly in the regulation of organic processes such as neuromuscular excitability, secretory processes, release of hormones, and neurotransmitters, besides the maintenance and formation of the bone matrix. In these situations, it acts as a transmitter of signs or as a protein activator. Maintaining calcium homeostasis is essential to the correct functioning of the body and also contributes to the better functioning of the other physiological systems [40,41,43].

In our study, calcium in Class I decreased at admission, increased at 48 hours, and decreased at 72 hours. Regarding Class II, calcium decreased at admission, increased at 48 hours, and normalized at 72 hours. Salivary calcium increased in relation to the normality pattern and, in relation to the times, it increased at 48 hours in Class II individuals compared to Class I. Due to their varied traumas and complex treatment, the individuals participating in this study may develop diseases that result in hypocalcemia or hypercalcemia. The calcium values presented here are in accordance with the literature. A decrease in calcium concentration after a trauma situation is associated with the fact that this individual will present hemodynamic changes, due to large volume losses, musculoskeletal trauma followed by fractures and stress, as well as vigorous replacements culminating with imbalances of the alkalosis type. In a study performed with trauma victims who presented a volume loss, serum calcium values behaved in a similar manner to that found, even when receiving adequate resuscitation when hospitalized (decrease, increase, stabilization with normality) [69].

In relation to the values found for salivary calcium, certain hypotheses may be proposed in order to explain this marked increase. The first is the high degree of stress present in the patients participating in the study, since they had suffered polytrauma. This higher salivary level of cortisol was verified in a previous study in adults in ICU with cranial trauma [23]. The autonomic nervous system regulates the salivation process, including the flow and concentration of certain salivary components, which provides a reliable measure of the sympathetic response [70]. Salivary flow can be altered by olfactory stimuli, exposure to luminosity, body position, and circadian cycle [14,15]. Another factor in the high level of calcium to be explained is that the intracellular calcium vesicles that act as second messenger, regulating various functions and being released on a strong hormonal stimulus, would be released in the salivary secretion because the patients are under a strong stimulus stressor [71]. Hormonal changes can have profound effects on metabolic homeostasis and circulatory hemodynamics and in the mechanisms of renal homeostasis and gastrointestinal physiology [72].

In addition to all the aforementioned functions, calcium, along with other minerals, promotes the maintenance of the isoelectric point of proteins, bone mineralization, transmission of nerve impulses, maintenance of the contraction mechanism and relaxation of the muscles, and also regulation of the ionic balance effect in enamel remineralization [52,65], thus guaranteeing temporary oral health in the patient. Because it is a transient state of high calcium, it also increases individuals’ susceptibility to dental calculus formation.

## Conclusion

This study provides evidence that salivary components can be used to follow clinical evolution in polytrauma patients admitted to the ICU.

## Supporting information

S1 Text Authorization of the Cajuru University Hospital. (PDF)

S2 Text Authorization for ICU study. (PDF)

S3 Text Consubstantiated opinion of CEP. (PDF)

S4 Text Free and informed consent form. (PDF)

S5 Text APACHE II Instrument. (PDF)

S6 Text Predictive mortality. (PDF)

S7 Text Coefficient of lethality. (PDF)

S8 Text Predictive value of APACHE II (APACHE II groups). (PDF)

S9 Text Data collection instrument. (PDF)
