# Supplementary material for: Sialochemical analysis in polytraumatized patients in intensive care units
Source: PLoS One. 2019 Oct 3;14(10):e0222974. doi: 10.1371/journal.pone.0222974 (PMC6776458; doi:10.1371/journal.pone.0222974)
Supplement: S1 Text — (PDF) [file pone.0222974.s001.pdf]

AUTORIZAÇÃO

Eu, Diretora Técnica: **DRª MARIA INÊS RAMINA** abaixo assinado, responsável pela(o)...**HOSPITAL UNIVERSITÁRIO CAJURU**, autorizo a realização do estudo: **AVALIAÇÃO SIALOQUÍMICA E SIALOMÉTRICA EM PACIENTES POLITRAUMATIZADOS EM UNIDADES DE TRATAMENTO INTENSIVO**, a ser conduzido pelos pesquisadores abaixo relacionados. Fui informado pelo responsável do estudo sobre as características e objetivos da pesquisa, bem como das atividades que serão realizadas na instituição a qual represento.

Declaro ainda ter lido e concordar com o parecer ético emitido pelo CEP da instituição proponente, conhecer e cumprir as Resoluções Éticas Brasileiras, em especial a Resolução CNS 196/96. Esta instituição está ciente de suas co-responsabilidades como instituição co-participante do presente projeto de pesquisa e de seu compromisso no resguardo da segurança e bem-estar dos sujeitos de pesquisa nela recrutados, dispondo de infra-estrutura necessária para a garantia de tal segurança e bem-estar.

Curitiba, 17 de [illegible] de 2015

Hospital Universitário Cajuru
Dra. Maria Inês C. Lorusso Ramina
CRM 21.382 – Diretora Técnica

Assinatura e carimbo do responsável institucional

LISTA NOMINAL DE PESQUISADORES:

MARIA HELOISA MADRUGA CHAVES
ALINE CRISTINA BATISTA RODRIGUES JOHANN
JOÃO ARMANDO BRANCHER
SUELEN TEIXEIRA LUIZ
ERICSON PEREIRA
