# Supplementary material for: Sialochemical analysis in polytraumatized patients in intensive care units
Source: PLoS One. 2019 Oct 3;14(10):e0222974. doi: 10.1371/journal.pone.0222974 (PMC6776458; doi:10.1371/journal.pone.0222974)
Supplement: S2 Text — (PDF) [file pone.0222974.s002.pdf]

ANEXO B - AUTORIZAÇÃO

Eu, **LUANA ALVES TANNOUS**, abaixo assinado, responsável pelo Serviço de Terapia Intensiva do Hospital Universitário Cajuru – Curitiba-PR, autorizo a realização do estudo: **AVALIAÇÃO SIALOQUÍMICA E SIALOMÉTRICA EM PACIENTES POLITRAUMATIZADOS EM UTI**, a ser conduzidos pelos pesquisadores abaixo relacionados.

Fui informado pelo responsável do estudo sobre as características e objetivos da pesquisa, bem como das atividades que serão realizadas na instituição a qual represento.

Declaro ainda ter lido e concordar com o parecer ético emitido pelo CEP da instituição proponente, conhecer e cumprir as Resoluções Éticas Brasileiras, em especial a Resolução CNS 196/96. Esta instituição está ciente de suas co-responsabilidades como instituição co-participante do presente projeto de pesquisa e de seu compromisso no resguardo da segurança e bem-estar dos sujeitos de pesquisa nela recrutados, dispondo de infra-estrutura necessária para a garantia de tal segurança e bem-estar.

Curitiba, 15 de [illegible] de 2015

Dra. Luana Alves Tannous
Intensivista
CRM PR 20.464

____

Assinatura e carimbo do responsável institucional

____

Maria Heloisa Madruga Chaves – (41) 98074388

____

Aline Cristina Batista Rodrigues Johann – (41) 84092406
