# Supplementary material for: Sialochemical analysis in polytraumatized patients in intensive care units
Source: PLoS One. 2019 Oct 3;14(10):e0222974. doi: 10.1371/journal.pone.0222974 (PMC6776458; doi:10.1371/journal.pone.0222974)
Supplement: S3 Text — (PDF) [file pone.0222974.s003.pdf]

PARECER CONSUBSTANCIADO DO CEP

DADOS DO PROJETO DE PESQUISA

Título da Pesquisa: AVALIAÇÃO SIALOQUÍMICA E SIALOMÉTRICA EM PACIENTES POLITRAUMATIZADOS EM UNIDADES DE TRATAMENTO INTENSIVO

Pesquisador: MARIA HELOISA MADRUGA CHAVES

Área Temática:

Versão: 2

CAAE: 51055515.3.0000.0020

Instituição Proponente: Pontifícia Universidade Católica do Parana - PUCPR

Patrocinador Principal: Financiamento Próprio

DADOS DO PARECER

Número do Parecer: 1.358.439

Apresentação do Projeto:

O crescimento descontrolado das cidades brasileiras bem como a marginalização de sua população, disparidades de condições sociais e estilos de vida caracterizam-na como uma sociedade conflituosa com grande número de violências e acidentes de trânsitos, características estas que representam um problema de saúde pública, sendo o trauma uma consequência relevante. O atendimento a pacientes oriundos de trauma ocorre na unidade de Terapia Intensiva (UTI), pois este local destina-se aos pacientes com quadros de saúde graves que necessitam de vigilância contínua e suporte terapêutico especializado. Atualmente o controle da evolução clinica destes pacientes é realizada por meio de exame físico, o qual é realizado pela equipe multiprofissional que atua no ambiente de UTI, bem como exame de imagem e laboratorial. Os procedimentos laboratoriais mais utilizados com fins diagnósticos envolvem a análise dos constituintes químicos e celulares do sangue. Os métodos de avaliação sérica são na sua maioria invasivos e apresentam custo elevado bem como causam dor. Associado a isso, o fato desses pacientes serem instáveis no que diz respeito à manutenção da saúde, os mesmos ficam mais susceptíveis a riscos de iatrogênicas e possíveis infecções. É neste contexto que algumas pesquisas têm avaliado o potencial da saliva como fluido biológico útil nos exames para diagnóstico de doenças sistêmicas ou localizadas na boca. O fato de a saliva poder ser facilmente

coletada quando comparada à coleta de sangue, tem despertado especial interesse nos pesquisadores.

Objetivo da Pesquisa:

Objetivo Primário:

Avaliar os parâmetros sialoquímicos e sialométricos no inicio e no final da internação de pacientes politraumatizados da UTI geral de um Hospital Universitário de Curitiba – PR.

Objetivo Secundário:

Comparar os parâmetros salivares, séricos e bacterianos no inicio e no final da internação; Correlacionar esses parâmetros; Correlacionar esses parâmetros com os dados dos prontuários desses pacientes.

Avaliação dos Riscos e Benefícios:

Os riscos e benefícios da pesquisa ao participante do estudo foram apresentados e justificados no projeto e estão em conformidade com a Res. 466/12.

Comentários e Considerações sobre a Pesquisa:

A pesquisa está relatada de maneira adequada e compreensível, não havendo qualquer necessidade de outros esclarecimentos.

Considerações sobre os Termos de apresentação obrigatória:

Todos os termos necessários para a realização do projeto foram apresentados e estão em conformidade com a Res. 466/12 - TCLE, TCUD e Carta de Autorização.

Recomendações:

A Resolução vigente para os CEP no território Nacional é a 466/12, favor alterar no projeto original.

O TCUD deve ser assinado pelos pesquisadores e depois disso digitalizados e encaminhados a secretaria deste CEP.

Conclusões ou Pendências e Lista de Inadequações:

O presente projeto de pesquisa encontra-se aprovado no quesito ético.

Considerações Finais a critério do CEP:

Lembramos aos senhores pesquisadores que, no cumprimento da Resolução 466/2012, o Comitê de Ética em Pesquisa (CEP) deverá receber relatórios anuais sobre o andamento do estudo, bem

Endereço: Rua Imaculada Conceição 1155
Bairro: Prado Velho **CEP:** 80.215-901
UF: PR **Município:** CURITIBA
Telefone: (41)3271-2103 **Fax:** (41)3271-2103 **E-mail:** nep@pucpr.br

como a qualquer tempo e a critério do pesquisador nos casos de relevância, além do envio dos relatos de eventos adversos, para conhecimento deste Comitê. Salientamos ainda, a necessidade de relatório completo ao final do estudo.

Eventuais modificações ou ementas ao protocolo devem ser apresentadas ao CEP-PUCPR de forma clara e sucinta, identificando a parte do protocolo a ser modificado e as suas justificativas.

Se a pesquisa, ou parte dela for realizada em outras instituições, cabe ao pesquisador não iniciá-la antes de receber a autorização formal para a sua realização. O documento que autoriza o início da pesquisa deve ser carimbado e assinado pelo responsável da instituição e deve ser mantido em poder do pesquisador responsável, podendo ser requerido por este CEP em qualquer tempo.

Este parecer foi elaborado baseado nos documentos abaixo relacionados:

Tipo Documento	Arquivo	Postagem	Autor	Situação
Informações Básicas do Projeto	PB_INFORMAÇÕES_BÁSICAS_DO_PROJETO_624790.pdf	03/12/2015 17:17:58		Aceito
Outros	TCUD.docx	03/12/2015 17:17:17	MARIA HELOISA MADRUGA CHAVES	Aceito
TCLE / Termos de Assentimento / Justificativa de Ausência	TCLE.docx	03/12/2015 15:49:40	MARIA HELOISA MADRUGA CHAVES	Aceito
Folha de Rosto	Folha_de_rosto.pdf	17/11/2015 15:31:30	MARIA HELOISA MADRUGA CHAVES	Aceito
Projeto Detalhado / Brochura Investigador	PROJETO.pdf	17/11/2015 15:25:53	MARIA HELOISA MADRUGA CHAVES	Aceito
Outros	COMITE.doc	16/11/2015 14:20:11	MARIA HELOISA MADRUGA CHAVES	Aceito

Situação do Parecer:
Aprovado

Necessita Apreciação da CONEP:
Não

Endereço: Rua Imaculada Conceição 1155
Bairro: Prado Velho **CEP:** 80.215-901
UF: PR **Município:** CURITIBA
Telefone: (41)3271-2103 **Fax:** (41)3271-2103 **E-mail:** nep@pucpr.br
