# Supplementary material for: Sialochemical analysis in polytraumatized patients in intensive care units
Source: PLoS One. 2019 Oct 3;14(10):e0222974. doi: 10.1371/journal.pone.0222974 (PMC6776458; doi:10.1371/journal.pone.0222974)
Supplement: S4 Text — (PDF) [file pone.0222974.s004.pdf]

**TERMO DE CONSENTIMENTO LIVRE E ESCLARECIDO (TCLE)**

Eu,.......................................................................................................................;
RG:....................................................................... **estou autorizando** a realização da coleta de saliva de:......................................................................., o qual se encontra internado na UTI geral do Hospital Universitário Cajuru como voluntário(a) a participar do estudo **AVALIAÇÃO SIALOQUÍMICA E SIALOMÉTRICA EM PACIENTES POLITRAUMATIZADOS EM UNIDADES DE TRATAMENTO INTENSIVO** e que tem como objetivo geral: Avaliar os parâmetros da saliva e sangue (sialoquímicos e sialométricos) no inicio e no final da internação de pacientes politraumatizados da UTI geral de um Hospital Universitário de Curitiba – PR; e como objetivos específicos: Comparar os parâmetros salivares, séricos e bacterianos no inicio e no final da internação; Correlacionar esses parâmetros; Correlacionar esses parâmetros com os dados dos prontuários desses pacientes. Acreditamos que ela seja importante porque: A relativa facilidade e a natureza não invasiva dos procedimentos de coleta da saliva tornam este fluído biológico um meio interessante para o desenvolvimento de pesquisas que visem seu emprego na realização de diagnósticos clínicos, e diante da escassez de estudos que avaliem as características da saliva e do sangue (sialoquímicas e sialométricas) em pacientes politraumatizados em UTI Geral, da relevância da avaliação sérica com relação da saúde do indivíduo, e da possível correlação entre os parâmetros séricos e salivares, este estudo justifica-se. Futuramente, esse estudo possivelmente poderá servir de ferramenta auxiliar para avaliação desses indivíduos.

**PARTICIPAÇÃO NO ESTUDO**

A minha participação no referido estudo será de autorizar a divulgação dos resultados dos exames da saliva e do sangue (sialometricos e sialoquimicos), do paciente acima citado. A coleta de saliva total acontecera em 3 momentos: na admissão, 48 e 72 horas após internação. A mesma será realizada posicionando-se o aspirador mecânico no assoalho lingual, na face lingual dos pré-molares. A ponta do sugador será movida do pré-molar de um dos lados em direção ao outro, passando pelo assoalho lingual na região de pré-molares, caninos e incisivos centrais e laterais. O movimento será repetido durante 5 minutos ininterruptos. A amostra será coletada no próprio leito da UTI.
A saliva coletada será armazenada em frasco coletor que estará acoplada ao aspirador e o volume de saliva coletado será estimado em mililitros por minuto.

**RISCOS E BENEFÍCIOS**

Fui alertado de que, da pesquisa a se realizar, posso esperar alguns benefícios para o meu representado, tais como: a identificação de alterações fisiológicas que culminem com o agravamento do estado de saúde, a diminuição da "dor" durante a coleta de material por não se tratar de uma pratica invasiva; conhecendo as

RÚBRICA DO SUJEITO DE PESQUISA

RÚBRICA DO PESQUISADOR

alterações da saliva e do sangue, resultara em maior compreensão dos processos patológicos (das doenças) que ocorrem neste paciente durante a internação na UTI, gerando subsídios para o desenvolvimento de futuros protocolos para o tratamento e acompanhamento dos mesmos além de permitir o conhecimento das variáveis que interferem na saúde.

Não há riscos para o paciente, a coleta não será feita por meio de processo invasivo, não há risco de dor e nem a necessidade de mobilizar o paciente para outro ambiente. A coleta não vai alterar o quadro clinico do mesmo. A amostra de sangue não será coletado pela pesquisadora e sim será coletada na rotina de exames da UTI.

Quando se detectado alterações (ressecamento da mucosa) durante a coleta da amostra, os mesmos serão então reavaliados pela equipe multidisciplinar que compõe o quadro da UTI. E também pela cirurgiã dentista responsável, afim de verificar a integridade da mucosa. E caso necessário acontecerá o tratamento odontológico.

A amostra de saliva será coletada em 3 momentos: na admissão, 48 e 72 horas após internação. A mesma será realizada posicionando-se o aspirador mecânico com sonda de aspiração de ponta flexível na boca, O movimento será repetido durante 5 minutos ininterruptos.

**SIGILO E PRIVACIDADE**

Estou ciente de que a privacidade do meu representado será respeitada, ou seja, nome ou qualquer outro dado ou elemento que possa, de qualquer forma identificar será mantido em sigilo. Os pesquisadores se responsabilizam pela guarda e confidencialidade dos dados, bem como a não exposição dos dados de pesquisa.

**AUTONOMIA**

É assegurada a assistência durante toda pesquisa, bem como me é garantido o livre acesso a todas as informações do meu representado e esclarecimentos adicionais sobre o estudo e suas consequências, enfim, tudo o que eu queira saber antes, durante e depois da participação do meu representado. Também fui informado de que posso recusar a participação do meu representado no estudo, ou retirar o consentimento a qualquer momento, sem precisar justificar, e de, por desejar sair da pesquisa, este não sofrerá qualquer prejuízo à assistência que vem sendo recebida.

RÚBRICA DO SUJEITO DE PESQUISA

RÚBRICA DO PESQUISADOR

**RESSARCIMENTO E INDENIZAÇÃO**

No entanto, caso ocorra qualquer despesa decorrente da participação do meu representado na pesquisa, tais como transporte, alimentação entre outros, haverá ressarcimento dos valores gastos na forma seguinte: em dinheiro, depositado em

conta corrente, caso não haja conta corrente o mesmo será entregue em espécie. De igual maneira, caso ocorra algum dano decorrente da participação do meu representado no estudo, serei devidamente indenizado, conforme determina a lei.

**CONTATO**

Os pesquisadores envolvidos com o referido projeto são: Maria Heloisa Madruga Chaves, Aline Cristina Batista Rodrigues Johann, João Armando Brancher, – PUCPR (Pontifícia Universidade Católica do Paraná) e com eles poderei manter contato pelos telefones: (41) 98074388; (41) 84092406; (41) 92036662, Suellen Teixeira Luiz (.41) 881771229; Ericson Pereira (41) 88149845

O Comitê de Ética em Pesquisa em Seres Humanos (CEP) é composto por um grupo de pessoas que estão trabalhando para garantir que seus direitos como participante de pesquisa sejam respeitados. Ele tem a obrigação de avaliar se a pesquisa foi planejada e se está sendo executada de forma ética. Se você achar que a pesquisa não está sendo realizada da forma como você imaginou ou que está sendo prejudicado de alguma forma, você pode entrar em contato com o Comitê de Ética em Pesquisa da PUCPR (CEP) pelo telefone (41) 3271-2292 entre segunda e sexta-feira das 08h00 as 17h30 ou pelo e-mail nep@pucpr.br.

RÚBRICA DO SUJEITO DE PESQUISA

RÚBRICA DO PESQUISADOR

**DECLARAÇÂO**

Declaro que li e entendi todas as informações presentes neste Termo de Consentimento Livre e Esclarecido e tive a oportunidade de discutir as informações deste termo. Todas as minhas perguntas foram respondidas e eu estou satisfeito com as respostas. Entendo que receberei uma via assinada e datada deste documento e que outra via assinada e datada será arquivada nos pelo pesquisador responsável do estudo.
Enfim, tendo sido orientado quanto ao teor de todo o aqui mencionado e compreendido a natureza e o objetivo do já referido estudo, manifesto meu livre consentimento para autorizar a participação do meu representado, estando totalmente ciente de que não há nenhum valor econômico, a receber ou a pagar, por minha participação.

| **Dados do participante da pesquisa** | |
|---|---|
| Nome: | |
| Idade: | |

| **Dados do represável pelo participante da pesquisa** | |
|---|---|
| Nome: | |
| Telefone: | |

Local, _____ de _____________ de _____.

Assinatura do participante da pesquisa

Assinatura do Pesquisador

Assinatura do responsável legal do participante da pesquisa

**USO DE IMAGEM**

**Não haverá uso de imagem.**

Assinatura do responsável legal do representado da pesquisa

Assinatura do Pesquisador

RÚBRICA DO SUJEITO DE PESQUISA

RÚBRICA DO PESQUISADOR
