# Supplementary material for: Sialochemical analysis in polytraumatized patients in intensive care units
Source: PLoS One. 2019 Oct 3;14(10):e0222974. doi: 10.1371/journal.pone.0222974 (PMC6776458; doi:10.1371/journal.pone.0222974)
Supplement: S5 Text — (PDF) [file pone.0222974.s005.pdf]

a) ESCORE FISIOLÓGICO AGUDO

**Variáveis fisiológicas**	+4	+3	+2	+1	0	+1	+2	+3	+4
Temperatura retal (C)	>41	39-40,9		38,5-38,9	36-38,4	34-35,9	32-33,9	30-31,9	< 29,9
Pressão arterial média mmHg	>160	139-159	110-129		70-109		50-69		< 40
Frequência cardíaca bpm	>180	140-179	110-139		70-109	55-69	40-54	< 39	
Frequência respiratória irpm (ventilados ou não)	>50	35-49	25-34	12-24	10-11	6-9		< 5	
Oxigenação A-aDO2									
a) FiO2 > 0,5 A-aDO2	>500	350-499	200-349		< 200				
b) FiO2< 0,5 PaO2					>70	61-70		55-60	< 55
pH Arterial	>7,7	7,6-7,69		7,5-7,59	7,33-7,49		7,25-7,32	7,15-7,24	< 7,15
Sódio sérico (mEq/L)	> 180	160-179	155-159	150-154	130-149		120-129	111-119	< 110
Potássio sérico (mEq/L)	> 7	6-6,9		5,5-5,9	3,5-5,4	3-3,4	2,5-2,9		< 2,5
Creatinina sérica (mg/dL) dobrar pontos se IRA	> 3,5	2-3,4	1,5-1,9		0,6-1,4		< 0,6		
Hematócrito (%)	> 60		50-50,9	46-49,9	30-45,9		20-29,9		< 20
Número de leucócitos	> 40		20-39,9	15-19,9	3-14,9		1-2,9		< 1
Escala de Glasgow para o coma Escore = (15-escore atual)									
Total do escore fisiológico agudo									
Bicarbonato sérico (mEq/L) (usar se não coletar gasometria)	> 52	41-51,9		32-40,9	22-31,9		18-21,9	15-17,9	< 15

b) PONTOS PARA A IDADE

Pontos	0	2	3	5	6
idade (anos)	< 44	45-54	55-64	65-74	> 75

c) PONTOS PARA DOENÇA CRÔNICA

Se o paciente tem uma história de insuficiência grave de órgãos ou é imunocomprometido; assinale pontos como se segue:

a) Para pacientes não-cirúrgicos ou pós-operatórios de emergência: 5 pontos

b) Para pacientes de pós-orperatórios eletivos: 2 pontos

Definições: a insuficiência de órgão ou o estado de imunodepressão deve ser evidente antes da admissão hospitalar e deve obedecer o seguinte critério:

Fígado: Cirrose comprovada por biópsia, hipertensão portal documentada; episódios passados de hemorragia gastrintestinal atribuídos à hipertensão portal; episódios anteriores de insuficiência hepatíca, encefalopatia ou coma

Cardiovascular: New York Association classe IV

Respiratória: Doença crônica restritiva, obstrutiva ou vascular resultando em grave restrição ao exercício, isto é, incapaz de subir escadas ou fazer serviços domésticos; hipóxia crônica documentada, hipercapnia, policitemia secundária, hipertensão pulmonar grave (> 40 mmHg); dependência de prótese ventilatória

Renal: Recebendo diálise cronicamente

Imunocomprometido: Paciente tem reebido terapia que suprime a resistência à infecção, isto é, imunossupressores, quimioterapia, radioterapia, corticóides cronicamente ou recente em altas doses; doença que é suficientemente avançada

para suprimir a resistência à infecção, isto é, leucemia, linfoma, AIDS
**ESCORE APACHE II = A + B + C**
