# Supplementary material for: Sialochemical analysis in polytraumatized patients in intensive care units
Source: PLoS One. 2019 Oct 3;14(10):e0222974. doi: 10.1371/journal.pone.0222974 (PMC6776458; doi:10.1371/journal.pone.0222974)
Supplement: S6 Text — (PDF) [file pone.0222974.s006.pdf]

Escore (pontos)	Risco de mortalidade
0-4	aproximadamente 4 %
5-9	aproximadamente 8%
10-14	aproximadamente 15%
15-19	aproximadamente 25%
20-24	aproximadamente 40%
25-29	aproximadamente 55%
30-34	aproximadamente 75%
> 34	aproximadamente 85%
