# Supplementary material for: Sialochemical analysis in polytraumatized patients in intensive care units
Source: PLoS One. 2019 Oct 3;14(10):e0222974. doi: 10.1371/journal.pone.0222974 (PMC6776458; doi:10.1371/journal.pone.0222974)
Supplement: S7 Text — (PDF) [file pone.0222974.s007.pdf]

Coeficientes	
Pacientes não-operatórios	
**Insuficiência respiratória**	
Asma/alergia	-2,108
DPOC	-0,367
SDRA (SARA)	-0,215
Parada respiraória	-0,168
Aspiração/envenenamento/tóxico	-0,142
Embolia pulmonar	-0,3120
Infecção	0
Neoplasia	0,891
**Insuficiência cardiovascular:**	
Hipertensão	-1,798
Distúrbio do ritmo	-1,368
Insuficiência cardíaca congestiva	-0,424
Choque hemorrágico/hipovolemia	0,493
Doença coronariana arterial	-0,191
Sepse	0,113
Choque cardiogênico	-0,259
Aneurisma dissecante torácido ou abdominal	0,731
Parada cardíaca	0
**Trauma**	
Trauma múltiplo	-1,228
Trauma craniano	-0,517
**Neurológico**	
Desordens convulsiva	-0,584
Hemorragia cerebral	0,723
**Outras**	
Overdose de drogas	-3,353
Cetoacidose diabética	-1,507
Hemorragia gastrintestinal	0,334
**IMOS**	
Metabólico/renal	-0,885
Respiratório	-0,890
Neurológico	-0,759
Cardiovascular	0,470
Gastrintestinal	0,501
Pacientes pós-operatórios	
Trauma múltiplo	-1,684
Admissão devida à doença cardiovascular crônica	-1,376
Cirurgia vascular periférica	-1,315
Cirurgia cardíaca valvular	-1,261
Craniotomia por neoplasia	-1,245
Cirurgia renal por neoplasia	-1,204
Transplante renal	-1,042

Traumatismo craniano	-0,955
Cirurgia torácica por neoplasia	-0,802
Craniotomia por hemorragia cerebral	-0,788
Laminectomia e outras cirurgias da coluna vertebral	-0,699
Choque hemorrágico	-0,682
Hemorragia gastrintestinal	-0,617
Cirurgia gastrintestinal por neoplasia	-0,248
Insuficiência respiratória pós-cirurgia	-0,140
Perfuração/obstrução abdominal	0,060
Sepse	0,113
Parada cardíaca	0,393
Parada respiratória	-0,168
**Outros**	
Cardiovascular	- 0,797
Respiratório	-0,610
Gastrintestinal	-0,613
Metabólico/renal	-0,196
Neurológico	-1,150
