# Supplementary material for: Sialochemical analysis in polytraumatized patients in intensive care units
Source: PLoS One. 2019 Oct 3;14(10):e0222974. doi: 10.1371/journal.pone.0222974 (PMC6776458; doi:10.1371/journal.pone.0222974)
Supplement: S8 Text — (PDF) [file pone.0222974.s008.pdf]

Grupo 1	Grupo 2	Grupo 3	Grupo 4
A – 0-4 pontos	A – 10-14 pontos	A – 20-24 pontos	> 30 pontos
B – 5-9 pontos	B – 15-19 pontos	B – 25 – 29 pontos	
