# Supplementary material for: Sialochemical analysis in polytraumatized patients in intensive care units
Source: PLoS One. 2019 Oct 3;14(10):e0222974. doi: 10.1371/journal.pone.0222974 (PMC6776458; doi:10.1371/journal.pone.0222974)
Supplement: S9 Text — (PDF) [file pone.0222974.s009.pdf]

1. IDENTIFICAÇÃO DO PACIENTE

Nome:

Idade: ________________________anos.

Sexo: M ( ) F ( ) **Escolaridade:**

Profissão:

Data de entrada na UTI:

Raça:

Naturalidade:

Nacionalidade:

Endereço:

CEP: **Telefone:**

Nome do responsável pela internação:

1.1. Politraumatizado: ( ) sim ( ) não

1.2. Recebeu sangue ou hemoderivados: ( ) sim ( ) não (se sim, responda 1.3 e 1.4)

1.3. ( ) sangue total ( ) concentrado de hemácia ( ) plaquetas ( ) crio precipitado

1.4. ( ) 1 unidade; ( ) 2 unidades ( ) 3 unidades ( ) 4 unidades ( ) + de 4 unidades

2. MOTIVO DA INTERNAÇÃO

2.1 trauma de crânio ( ) sim ( ) não

2.1.1. ( ) Trauma craniano fechado

2.1.2.( ) Trauma craniano com abaulamento

2.1.3.( ) Fratura exposta de crânio

2.1.4 Tratamento realizado: ( ) cirúrgico ( ) clinico

2.1.5 Trauma de face ( ) sim ( ) não

2.1.6 Classificação das lesões na face: ( ) Fratura Nasal ( ) Fratura Alvéolo Dentaria ( ) Fratura de Mandíbula ( ) Fratura de Terço Médio da Face.

2.1.7 Tratamento realizado: ( ) cirúrgico ( ) clinico

2.2. Trauma de tórax: ( ) sim ( ) não

2.2.1. Classificação: ( ) aberto ( ) fechado

2.2.2. Quanto ao agente causal: ( ) FAF ( ) FAB ( ) Acidentes automobilísticos ( ) outros

2.2.3. Quanto a manifestação clínica: ( ) Pneumotórax {hipertensivo ou não} ( ) Hemotórax ( ) Tamponamento cardíaco ( ) Contusão pulmonar

( ) Lesão de grandes vasos { aorta; artéria pulmonar; veias cavas } ( ) outros.

2.3. Trauma de Abdômen: ( ) sim ( ) não

2.3.1. Classificação: ( ) aberto ( ) fechado

2.3.2. Trauma abdominal fechado: ( ) direto ( ) indireto

2.3.3. Trauma abdominal aberto: ( ) penetrante ( ) perfurante

2.4. Trauma de extremidades: ( ) sim ( ) não

2.4.1. Classificação: ( ) completa ( ) incompleta

2.4.2. Quanto ao foco: ( ) fechada ( ) aberta ou exposta

2.4.3. Local da lesão: ( ) membros superiores ( ) membros inferiores ( ) pelve

2.5. Medicamentos em uso

2.5.1 Sedação ( ) sim ( ) não	2.5.2 analgesia ( ) sim ( ) não
2.5.1.1 Midazolan ( )	2.5.2.1 Fentanil ( )
2.5.1.2 Propofol ( )	2.5.2.2 Morfina ( )
2.5.1.3 Diazepan ( )	2.5.2.3 Tramadol ( )

2.5.1.4 Haloperidol ( )
2.5.1.5 Tiopental ( )

2.6. Anticonvulsivante

2.6.1 Fenobarbital ( )
2.6.2 Hidantal ( )
2.6.3 Gardenal ( )
2.6.4 Fenitoína ( )
2.6.5 Sulfato de Magnésio ( )
2.6.6 Carbamazepina ( )

2.7. Medicamentos Protetores da mucosa gástrica

2.7.1. Antiácidos ( )sim ( )não
Bicarbonato de sódio ( )sim ( )não Hidróxido de alumínio ( ) sim ( ) não
Hidróxido de magnésio ( )sim ( )não Carbonato de cálcio ( )sim ( )não

2.7.2. Bloqueadores dos receptores histaminérgicos H2 ( )sim ( )não
Cimetidina ( )sim ( )não Ranitidina ( )sim ( )não Nizatidina ( ) sim ( )não

2.7.3. Bloqueadores da bomba de prótons ( )sim( )não
Omeprazol ( ) sim ( ) não Lanzoprazol ( ) sim ( )não Pantoprazol ( ) sim ( )não

2.8. Medicamentos antieméticos

2.8.1. Bromoprida	( ) sim ( )não
2.8.2. Digesam	( )sim ( )não
2.8.3 Dramin	( )sim ( )não
2.8.4. Plasil	( )sim ( )não
2.8.5. Plamet	( ) sim ( )não

2.9. Antibióticos: ( ) sim ( ) não

2.10. Microrganismo isolado:

Hemocultura	( ) sim ( ) não
Staphylococcus aureus	( ) sim ( ) não
Pseudomonas aeruginosa	( ) sim ( ) não
Escherichia coli	( ) sim ( ) não
Staphylococcus sp	( ) sim ( ) não
Enterobacter cloacae	( ) sim ( ) não
Streptococcus pneumoniae	( ) sim ( ) não
Streptococcus viridans	( ) sim ( ) não
Streptococcus haemoliticus	( ) sim ( ) não
Streptococcus pyogenes	( ) sim ( ) não
Streptococcus epidermidis	( ) sim ( ) não
Streptococcus sp	( ) sim ( ) não
Klebsiella pneumoniae	( ) sim ( ) não
Listeria sp	( ) sim ( ) não
Proteus vulgaris	( ) sim ( ) não
Enterobacter aerogenes	( ) sim ( ) não
Criptococcus sp	( ) sim ( ) não
Sem crescimento bacteriano	( ) sim ( ) não

2.11. Microrganismo isolado:

Secreção traqueal	( ) sim ( ) não

Pseudomonas aeruginosa	( ) sim ( ) não
Staphylococcus aureus	( ) sim ( ) não
Acinetobacter baumanii	( ) sim ( ) não
Proteus mirabilis	( ) sim ( ) não
Staphylococcus epidermidis	( ) sim ( ) não
Enterobacter cloacae	( ) sim ( ) não
Enterobacter aerogenes	( ) sim ( ) não
Staphylococcus sp	( ) sim ( ) não
Escherichia coli	( ) sim ( ) não
Acinetobacter calcoaceticus	( ) sim ( ) não
Klebsiella pneumoniae	( ) sim ( ) não
Enterococcus faecium	( ) sim ( ) não
Enterobacter aglomerans	( ) sim ( ) não
Sem crescimento bacteriano	( ) sim ( ) não

2.12. Microrganismo isolado:

Ponta de cateter venoso central	( ) sim ( )não
Staphylococcus aureus,	( ) sim ( )não
Staphylococcus epidermidis	( ) sim ( )não
Pseudomonas aeruginosa	( ) sim ( )não
Enterobacter cloacae,	( ) sim ( )não
Enterococcus faecalis.	( ) sim ( )não
Sem crescimento bacteriano	( ) sim ( )não

2.13. Microrganismo isolado:

Cultura de LCR (liquor)	( )sim ( )não
Enterobacter cloacae	( )sim ( )não
Proteus mirabilis	( )sim ( )não
Pseudomonas aeruginosa	( )sim ( )não
Staphylococcus aureus	( )sim ( )não
Escherichia coli	( )sim ( )não
Enterobacter aerogenes	( )sim ( )não
Sem crescimento bacteriano	( )sim ( )não

2.14. Classe dos antibióticos usados:

Penicilina	( )sim ( )não
Cefalosporina	( )sim ( )não
Monobactamas	( )sim ( )não
Anfenicóis	( )sim ( )não
Tetraciclinas	( )sim ( )não
Polipeptídios	( )sim ( )não
Poliênicos	( )sim ( )não
Macrolídios	( )sim ( )não
Aminoglicosídios	( )sim ( )não
Ansamicinas	( )sim ( )não
Antraciclinas	( )sim ( )não
Lincomicinas	( )sim ( )não
Nucleosídios;	( )sim ( )não
Glutarimidas	( )sim ( )não
Poliéter ionóforos	( )sim ( )não

2.14.1. Antibióticos em uso:	
Penicilina G.	( ) sim ( )não
Penicilina V	( ) sim ( )não
Meticilina	( ) sim ( )não
Oxacilina	( ) sim ( )não
Nafcilina	( ) sim ( )não
Ampicilina	( ) sim ( )não
Amoxicilina	( ) sim ( )não
Carbenicilina	( ) sim ( )não
Ticarcilina	( ) sim ( )não
Mezlocilina	( ) sim ( )não
Piperacilina	( ) sim ( )não

2.14.3. Antibióticos em uso:	
Astreonam	( )sim ( ) não
Sulfacetina	( ) sim ( ) não
Carumonam	( )sim ( ) não

2.14.4. Antibióticos em uso:	
Cloranfenicol	( )sim ( ) não
Tianfenicol	( )sim ( ) não

2.14.5. Antibióticos em uso:	
Tetraciclina	( )sim ( ) não
Doxiciclina	( )sim ( ) não
Minociclina	( )sim ( ) não

2.14.6. Antibióticos em uso:	
Actinomicina	( )sim ( ) não
Bacitracina	( )sim ( ) não
Colistina	( )sim ( ) não
Polimixina	( )sim ( ) não

2.14.7. Antibióticos em uso:	
andicidina	( )sim ( ) não
Natamicina	( )sim ( ) não
Sedamicina	( )sim ( ) não
Mocimicina	( )sim ( ) não

2.14.8. Antibióticos em uso:	
Eritromicina	( )sim ( ) não
Azitromicina	( )sim ( ) não
Claritromicina	( )sim ( ) não
Diritronicina	( )sim ( ) não
Roxitromicina	( )sim ( ) não

2.14.9. Antibióticos em uso:	
Amicacina	( )sim ( ) não
Arbecacina	( )sim ( ) não
Gentamicina	( )sim ( ) não
Canamicina,	( )sim ( ) não

Neomicina	( )sim ( ) não
Netilmicina	( )sim ( ) não
Paromomicina	( )sim ( ) não
Rodostreptomicina 1	( )sim ( ) não
Estreptomicina	( )sim ( ) não
Tobramicina	( )sim ( ) não
Apramicina	( )sim ( ) não
**2.14.10. Antibióticos em uso:**	
Rifamicina	( ) sim ( ) não
Rifamida	( ) sim ( ) não
Rifaximina	( ) sim ( ) não
Rifacetina	( ) sim ( ) não
**2.14.11. Antibióticos em uso:**	
Carubicina	( ) sim ( ) não
Pirarubicina	( ) sim ( ) não
Daurobimicina	( ) sim ( ) não
Doxorubicina	( ) sim ( ) não
Epirubicina	( ) sim ( ) não
**2.14.12. Antibióticos em uso:**	
Clindamicina	( ) sim ( ) não
Lincomicina	( ) sim ( ) não
Pirlimicina	( ) sim ( ) não
**2.14.13. Antibióticos em uso:**	
Amicelina	( ) sim ( ) não
Toiocamicina	( ) sim ( ) não
Tubercidina	( ) sim ( ) não
Tubercidina	( ) sim ( ) não
Puromicina	( ) sim ( ) não
**2.14.14. Antibióticos em uso:**	
Actifenol	( ) sim ( ) não
Cicloexamida	( ) sim ( ) não
**2.14.15. Antibióticos em uso:**	
Calcimicina	( ) sim ( ) não
Nigericina	( ) sim ( ) não
Maduramicina	( ) sim ( ) não
**2.15. Drogas vasoativas:**	
a) Noradrenalina	( ) sim ( ) não
b) Dobutamina	( ) sim ( ) não
c) Dopamina	( ) sim ( ) não
**2.16. Hormônio antidiurético**	
a) Vasopressina	( ) sim ( ) não
**2.17. Drogas vasodilatadoras**	

a) Nitroprussiato de sódio	( ) sim ( ) não
a) Lactulona	( )sim ( ) não
**2.18. Reguladores do transito intestinal ou laxante**	
a) Lactulona	( ) sim ( ) não
**3. Intervenções realizadas**	
a) Cânula orotraqueal	( )7,0;( )7,5;( )8,0;( )8,5;( )9,0;( )9,5;( )10,0
b) Traqueostomia	( ) sim ( ) não
c) Punção de subclávia	( ) sim ( ) não
d) Punção de jugular interna	( ) sim ( ) não
e) Punção de PAM	( ) sim ( ) não
f) Punção de acesso venoso periférico	( ) sim ( ) não
g) Punção de jugular externa	( ) sim ( ) não
h) Sondagem naso- gástrica	( ) sim ( ) não
i) Sondagem enteral	( ) sim ( ) não
j) Aspiração traqueal sistema fechado	( ) sim ( ) não
k) Aspiração traqueal sistema aberto	( ) sim ( ) não
l) Cateterismo vesical de alivio	( ) sim ( ) não
m) Cateterismo vesical de demora	( ) sim ( ) não
**3.1. Ventilação mecânica**	
4.1 ventilação controlada (CMV)	( ) sim ( ) não
4.2. ventilação assistida (AMV)	( ) sim ( ) não
4.3. ventilação assistida-controlada (A/C)	( ) sim ( ) não
4.4. ventilação mandatória intermitente (SIMV)	( ) sim ( ) não
4.5. ventilação de pressão de suporte (PSV)	( ) sim ( ) não
4.6. ventilação com pressão controlada(PCV)	( ) sim ( ) não
4.7. pressão continua nas vias aéreas (CPAP)	( ) sim ( ) não
**4. HISTORIA ODONTOLÓGICA**	
4.1. Número de dentes: ( )	
4.2.Uso de prótese: ( ) sim ( ) não	
4.3. Prótese total ( ) Prótese parcial ( )	
4.4. Local da prótese : ( ) superior ( ) inferior ( ) ambas	
4.5.Higiene oral com clorexedine: ( ) sim ( ) não	
4.6.Higiene oral realizada com creme dental: ( ) sim ( ) não	
4.7.Higiene oral é realizada quantas vezes a cada 12 hs: ( ) 1; ( ) 2; ( ) 3; ( ) 4; ( ) 6	
**4.1 Para higiene oral o que a equipe de saúde utiliza:**	
( ) escova dental	
( ) espátula e gaze	
( ) pinça e gaze	
( ) outro (especificar): ____	
